# Global depth perception alters local timing sensitivity

**DOI:** 10.1371/journal.pone.0228080

**Published:** 2020-01-23

**Authors:** Nestor Matthews, Leslie Welch, Elena K. Festa, Anthony A. Bruno, Kendra Schafer

**Affiliations:** 1 Department of Psychology, Denison University, Granville, OH, United States of America; 2 Department of Cognitive, Linguistic & Psychological Sciences, Brown University, Providence, RI, United States of America; Nottingham Trent University, UNITED KINGDOM

## Abstract

Dynamic environments often contain features that change at slightly different times. Here we investigated how sensitivity to these slight timing differences depends on spatial relationships among stimuli. Stimuli comprised bilaterally presented plaid pairs that rotated, or radially expanded and contracted to simulate depth movement. Left and right hemifield stimuli initially moved in the same or opposite directions, then reversed directions at various asynchronies. College students judged whether the direction reversed first on the left or right–a temporal order judgment (TOJ). TOJ thresholds remained similar across conditions that required tracking only one depth plane, or bilaterally synchronized depth planes. However, when stimuli required simultaneously tracking multiple depth planes–counter-phased across hemifields–TOJ thresholds doubled or tripled. This effect depended on perceptual set. Increasing the certainty with which participants simultaneously tracked multiple depth planes reduced TOJ thresholds by 45 percent. Even complete certainty, though, failed to reduce multiple-depth-plane TOJ thresholds to levels obtained with single or bilaterally synchronized depth planes. Overall, the results demonstrate that global depth perception can alter local timing sensitivity. More broadly, the findings reflect a coarse-to-fine *spatial* influence on how we sense *time*.

## Introduction

Motion can blind us to other visual features. For example, a recent wave of magician-inspired neuroscience [1–4] prompted the discovery that global linear motion reversals render local orientation changes invisible [5]. Likewise, local color changes readily seen in stationary stimuli disappear when the stimulus-set globally reverses between rotational motion directions [6] (See web demo at https://www.sciencedirect.com/science/article/pii/S0960982210016507?via%3Dihub). Both findings demonstrate motion-induced change blindness. In each case, global-direction reversals impaired sensitivity to local non-motion features, e.g., orientation and color. Here we investigated the ironic case of global-direction reversals impairing sensitivity to the timing of their constituent local-direction reversals.

**Fig 1:**
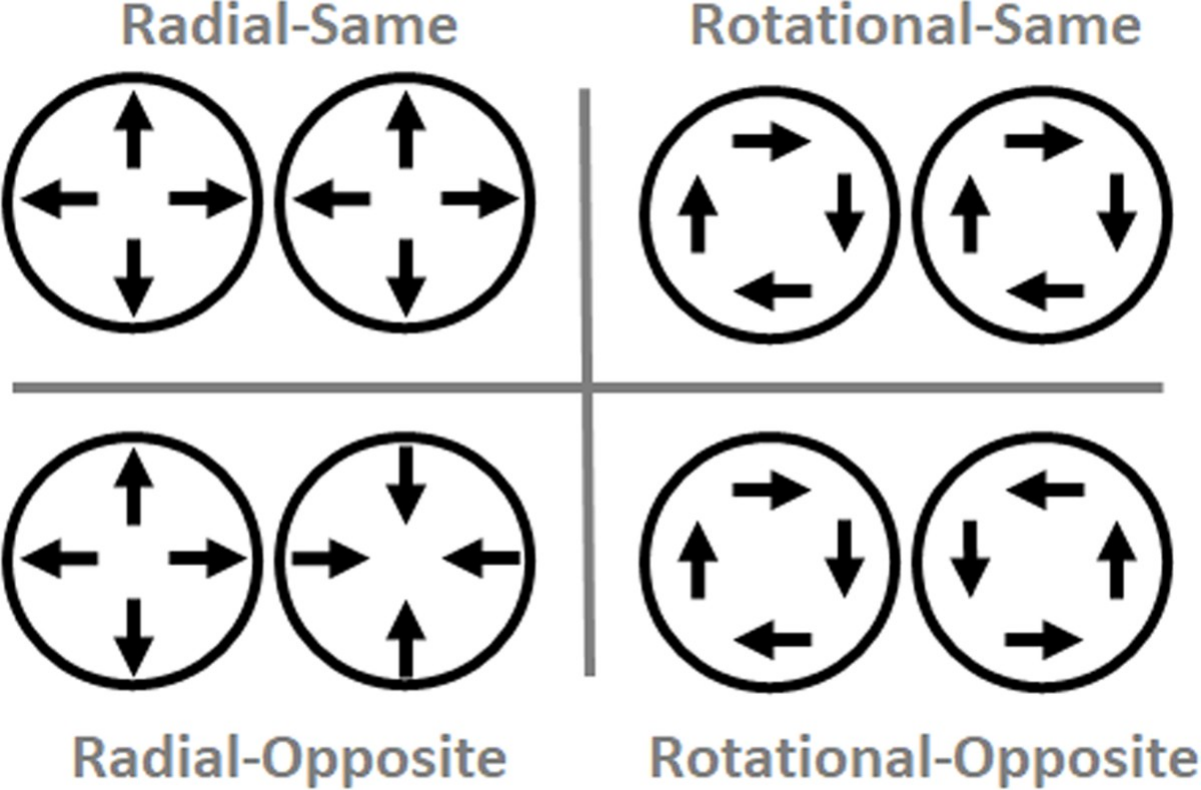
Schematic of stimuli from Matthews, Welch & Festa (2018). Stimuli comprised bilaterally presented dynamic plaid pairs. On each trial both plaids radiated (left) or rotated (right), and initially moved in the same (top) or opposite (bottom) directions before reversing directions. Participants judged whether the left or right plaid changed direction first–a temporal order judgment (TOJ). TOJ thresholds in the Radial-Opposite condition doubled or tripled relative to the other conditions, yet all conditions contained the same local linear motion components (arrows). Note that rotating the local linear motion components (arrows) 90° changes the emergent motion within each plaid from radial to rotational motion, and vice versa.

The impetus for this study came from a serendipitous finding in our lab [7]. The task required percussion, brass, and color guard drum corps experts to judge the temporal order of direction reversals in bilaterally presented plaid pairs. The two plaids within each pair moved radially or rotationally, and in the same initial direction or in opposite initial directions (see Fig 1). We had planned to investigate group differences in temporal order judgments (TOJs), as predicted by various theories. Although we found significant group differences, we also stumbled upon a larger effect. Within each group, the Radial-Opposite TOJ thresholds doubled or tripled compared to those in the other conditions.

Why would plaid pairs with radially opposite directions induce “blindness” to the temporal order of local-direction reversals? An insight comes from participants’ spontaneous reports about depth perceived in radially expanding and contracting stimuli. Specifically, numerous participants spontaneously described the Radial-Opposite stimuli as “…difficult because they move in and out”. Some participants also offered accompanying hand gestures that signaled counter-phase depth movement; one hand moved nearer in depth while the other moved away in depth. These spontaneous, informal verbal and non-verbal reports demonstrate that, in our Radial-Opposite condition, participants *perceived* global (cross-hemifield) counter-phase depth. Stated differently, changing the initial directions from same to opposite had qualitatively distinct perceptual consequences in the radial and rotational conditions. Radial-Opposite directions induced global (cross-hemifield) counter-phase depth percepts that did not occur in the other stimulus conditions. Therefore, global depth perception could have doubled or tripled TOJ thresholds uniquely in the Radial-Opposite condition.

The possibility that global (cross-hemifield) depth perception alters local (within hemifield) timing sensitivity framed the present study. In Experiment 1 we conducted a direct replication attempt to assess the reliability of the above-described, serendipitously-discovered doubling and tripling of Radial-Opposite TOJ thresholds [7]. In Experiment 2 we investigated how strongly this Radial-Opposite TOJ threshold elevation depends on three depth-related variables. These include depth uncertainty, common-fate motion in depth, and attentionally hastening the perception of stimuli looming in depth.

## Experiment 1 Direct replication: Materials and methods

The Denison University Human Participant Committee approved all experiments reported here, which we conducted with the written informed consent of each participant. The experiments adhere to the October 2008 Declaration of Helsinki. To promote reproducibility, the Open Science Framework [https://osf.io/d72bv/] contains the complete data set and all software necessary for replicating the experiment and the statistical analyses.

### Participants

Fifty-five college-aged undergraduates with normal or corrected vision participated in Experiment 1.

### Materials and apparatus

We used the apparatus and stimuli described in our recent study [7], and repeat those details here for completeness. The experiment ran on HP EliteOne 800 desktop computers, each with a Microsoft Windows 10 Enterprise operating system. Matlab 2017a software called functions from the psychophysics toolbox [8, 9]. We set the 23 inch flat screen HP LCD display’s resolution to 1920 × 1080 pixels, and the vertical refresh rate to 60 Hz. Although we did not stabilize head position, participants typically viewed the monitor from approximately 57 cm.

### Plaid stimuli

**Fig 2:**
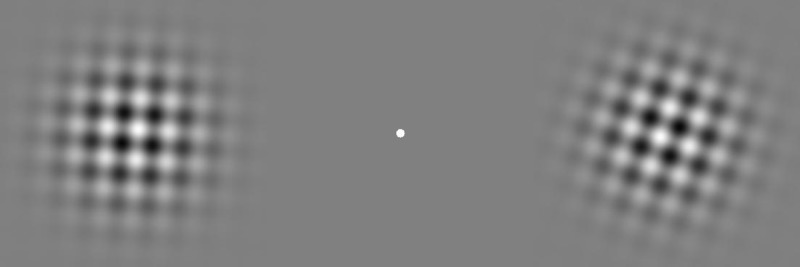
Sample movie frame. On each trial, a pair of bilaterally presented plaids either radiated or rotated. The plaids within each pair moved initially in either the same direction or opposite directions, randomly across trials. The two plaids subsequently changed direction, either at the same time or at various asynchronies. Participants indicated whether the left plaid or right plaid changed direction first—a TOJ.

On each trial participants viewed bilaterally presented dynamic plaid stimuli, shown in a 1-s (60-frame) movie. A sample frame from one movie appears in Fig 2. We centered each plaid 9.7° left or right of a white fixation point (152 cd/m^2^) in a gray surround (32.5 cd/m^2^). Each plaid had 95.47% Michelson contrast, a two-dimensional Gaussian window, and a 9.7° diameter. Within each plaid the two component gratings had identical spatial frequencies and identical spatial phases. The component spatial frequencies ranged randomly across approximately three octaves (0.25–1.9 cycles/°), and the spatial phases ranged randomly across 360°. One of the two component orientations on each trial ranged randomly across 180°, and the other differed by 90° from that randomly selected orientation.

### Radial and rotational speeds & direction changes

On each trial, the left and right plaid-component-gratings either shared a radial speed of two octaves per second or shared a rotational speed of 0.102 rotations per second. Those radial and rotational speeds generated comparable local linear speed gradients, which ranged between zero and 3.114° / sec. Each plaid initially moved in one radial or rotational direction for 333–666 ms, then reversed to the opposite direction for the remaining 333–666 ms. The direction reversals in the left and right plaids occurred either synchronously, or at ±67, ±133, or ±200 ms asynchronies. For illustration and ease of viewing, the Supporting Information contains sample movies (S9–S14 Movies) that display 300 ms asynchronies.

### Task

Participants pressed either the left or right arrow key to signal whether the left or right plaid changed direction first–a TOJ. The LCD monitor visually displayed immediate accuracy feedback after each response.

### Procedure

To develop familiarity with the dynamic plaid stimuli, participants began the experiment passively viewing movies containing relatively exaggerated (300 ms) asynchronies. (See Supporting Information.) Participants then completed a practice block of 112 TOJ trials, separated into four block-randomized 28-trial sets. Each 28-trial set comprised two Motion-Types (radial, rotational) crossed with two Initial-Directions (same, opposite) and seven temporal asynchronies (0, ±67, ±133, or ±200 ms). Negative (“left lagging”) and positive (“left leading”) asynchronies corresponded respectively to trials on which the right plaid and left plaid changed direction first. When the left and right plaids changed direction simultaneously (0 asynchrony trials) the computer pre-designated the correct response as “left first” or “right first” with equal probability. This neutral feedback on 0-asynchrony trials allowed us to assess each participant’s “left-first” versus “right-first” TOJ response bias [10].

After the 112-trial practice block, participants completed five additional 112-trial blocks (560 total trials) for analysis. These 112-trial blocks matched the practice block in all ways, including the four block-randomized 28-trial sets. Between the 112-trial blocks, each participant rested for 30 seconds while the computer displayed the participant’s cumulative percentage of correct responses. The experiment typically required about 25 minutes.

### Research design

We administered the independent variables via a 2 x 2 (Motion-Type x Initial-Directions) within-participant experimental research design. As noted above, the computer block-randomly manipulated the within-participant independent variables: Motion-Type (radial, rotational) and Initial-Directions (same, opposite). Control variables included counter-balancing which plaid (left, right) changed direction first, and the temporal asynchrony of the direction changes (0, ±67, ±133, or ±200 ms). We measured TOJ precision as the dependent variable.

### Statistical analyses: Signal detection theory

For each participant, we used standard procedures from Signal Detection Theory [11] to evaluate time sensitivity, i.e., TOJ precision indexed by d′. Operationally, hits and false alarms occurred respectively when participants made “left first” responses and the left plaid changed direction first or second. Computationally, we determined each participant's d′ value using the formula d′ = Z_Hits_ − Z_FalseAlarms_, with the Z-distribution's SD = 0.5. Accordingly, d′ = 0.67 corresponded to non-biased 75% correct performance.

For each participant we computed four d’ values, one for each (2x2) combination of the Motion-Type and Initial-Directions variables. For each d’ value we pooled *across* negative asynchronies to determine Z_FalseAlarms_ and positive asynchronies to determine Z_Hits_. Because z-transformations require proportions greater than zero and less than one, we adopted the following procedure from Stanislaw & Todorov (1999) [12]. For participants achieving 0 / 60 false alarms we assumed 0.5 / 60 false alarms. Conversely, for participants achieving 60/60 hits we assumed 59.5 / 60 hits. Note that pooling temporal asynchronies to estimate TOJ sensitivity (d’) parallels how a psychometric function pools temporal asynchronies to estimate TOJ thresholds.

### Statistical analyses: Psychometric functions & thresholds

We further evaluated TOJ precision by constructing four psychometric functions, one for each (2x2) combination of the Motion-Type and Initial-Directions variables. For a given combination of Motion-Type and Initial-Directions, the psychometric function's ordinate reflected the participants’ median proportion of “left-first” responses. The abscissa comprised the seven asynchronies, which ranged between −200 (“left lagging”) and +200 (“left leading”) ms, in ~67-ms steps. A least-squares procedure fit the data with the following sigmoidal function:

$$\frac{1}{1+exp(-K\;(X-Xo))}$$

K and Xo determine the sigmoid’s slope and midpoint, respectively. In each case, Pearson correlations indicated that the sigmoid significantly fit (p <0.001)–and explained more than 92% of–the response variability. The significant sigmoidal fits permitted estimating the 75% just noticeable difference, i.e., TOJ thresholds. We defined each TOJ threshold as half the stimulus change (temporal asynchrony) required to alter the “left-first” response rate from 0.25 to 0.75. Lower thresholds indicate better time sensitivity, i.e., finer TOJ precision.

For each threshold the following procedure generated error bars. First, we determined the standard error (SE) of each sigmoid estimate, expressed in the psychometric function’s y-axis units (proportion of “left-first” responses; centiles). The SE required computing the square root of the mean squared differences between each psychometric function’s observed and predicted (sigmoidal) values [13]. We then subtracted the SE from the observed value at each negative asynchrony, and added the SE to the observed value at each positive asynchrony. This steepened the slope of the observed values by one SE. Fitting a new sigmoid to this steepened data set generated a new threshold estimate one SE lower (finer) than that for the observed values. We repeated these steps after flattening the slope by adding and subtracting one SE to the observed values at negative and positive asynchronies, respectively. Fitting a another sigmoid to this flattened data set generated a threshold estimate one SE higher (worse) than that for the observed values. In short, this procedure converted y-axis units (centiles) to x-axis units (ms), and permitted TOJ threshold estimates one SE above and below the original estimate. The Open Science Framework [https://osf.io/d72bv/] contains the software for this procedure.

### Inclusion / exclusion criteria

The statistical analyses included data from each participant whose TOJ performance statistically exceeded chance (binomial test p<0.001). This inclusion criterion minimally required 57.5% correct TOJ performance across the 480 non-zero asynchrony trials for analysis. (The remaining 80 zero-asynchrony trials for analysis provided no basis for objectively determining a correct TOJ.) This criterion resulted in excluding data from 24 participants. Data from the remaining 31 (56.36% of 55) participants appear in the Results. As noted above, the Open Science Framework [https://osf.io/d72bv/] contains the raw data from *all* participants.

## Experiment 1: Results

### Replication of interaction effect on time sensitivity

**Fig 3:**
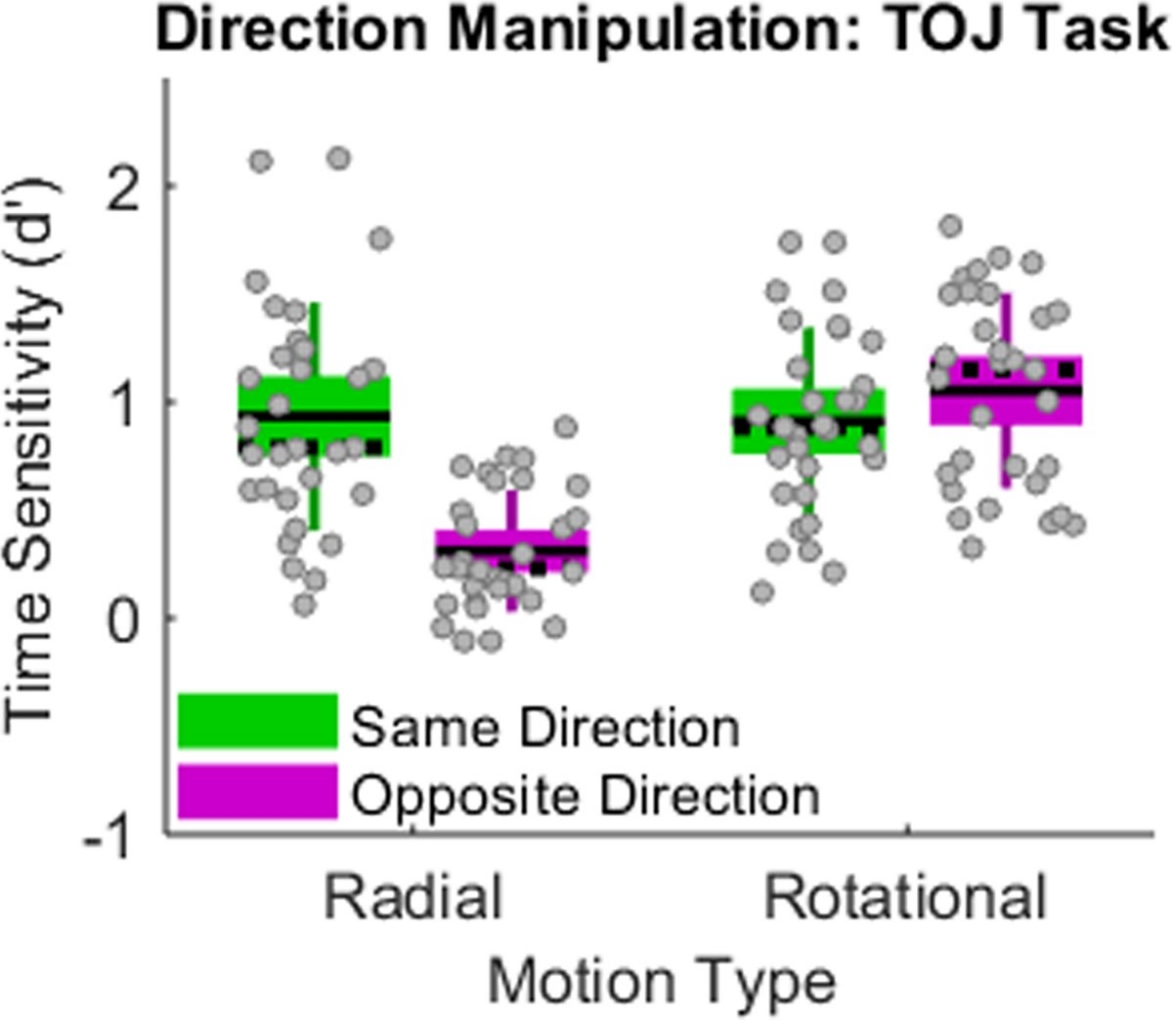
Motion type by initial directions interaction effect. Changing the initial directions from same (green boxes) to opposite (magenta boxes) impaired TOJ precision (d´) in the radial motion condition. This pattern reversed when the motion type changed to rotational motion. Unlike standard box plots, each box extends upward and downward by 1 SD from the mean—the solid horizontal line centered within each box. The dotted horizontal line indicates the median, and the error bars indicate the 95% confidence interval. Gray dots correspond to individual data points, which collectively satisfied the normalcy assumption in each condition (Lilliefors test).

Fig 3 illustrates a direct replication of the previously reported Motion-Type by Initial-Directions interaction [7]. Changing the initial directions from same (Fig 3, green boxes) to opposite (Fig 3, magenta boxes) impaired radial TOJs while modestly improving rotational TOJs. A multivariate 2x2 repeated-measures ANOVA confirmed that Motion-Type and Initial-Directions interacted significantly (F(1,30) = 67.324, p < 0.001, _p_eta^2^ = 0.692, power = 1.0).

**Fig 4:**
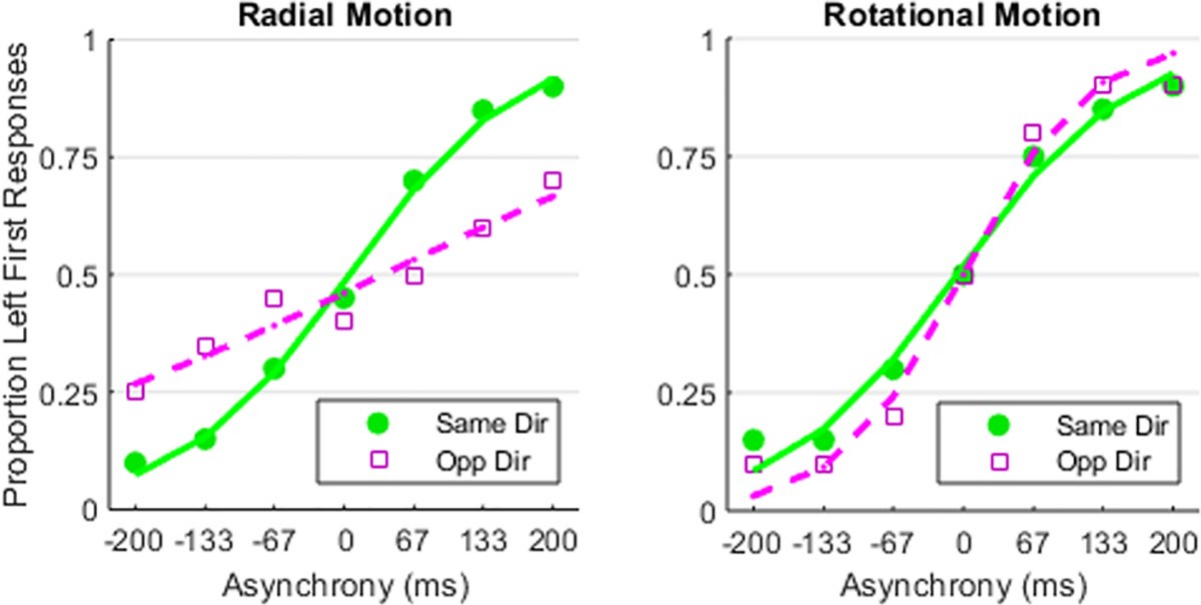
Psychometric functions. Solid green circles and trend lines indicate performance when the two stimuli initially moved in the same direction. Open magenta squares and dotted trend lines indicate performance when the two stimuli initially moved in opposite directions. Trend lines reflect the best fitting psychometric functions. Steeper slopes reflect better temporal precision (lower TOJ thresholds). Changing from same to opposite initial directions markedly impaired radial TOJs (left panel) and modestly improved rotational TOJs (right panel).

The significant interaction also appears readily in the slopes of Fig 4‘s psychometric functions. For radial TOJs (Fig 4, left panel), changing the initial directions from same (green circles) to opposite (magenta squares) markedly reduced the psychometric function’s slope. Visually inspecting Fig 4 reveals that this decreased slope reflects worse opposite-direction performance than same-direction performance at *each* asynchrony (±67, ±133, ±200 ms). Dissimilarly, for rotational TOJs (Fig 4, right panel), changing the initial directions from same to opposite modestly steepened the psychometric function’s slope. Despite these stimulus-specific differences in slope (precision), no corresponding differences emerged in the midpoints (accuracy) of the psychometric functions. Moreover, all midpoints tended toward the 0 ms asynchrony. This indicates that participants made left-first and right-first responses in a nonbiased manner.

**Fig 5:**
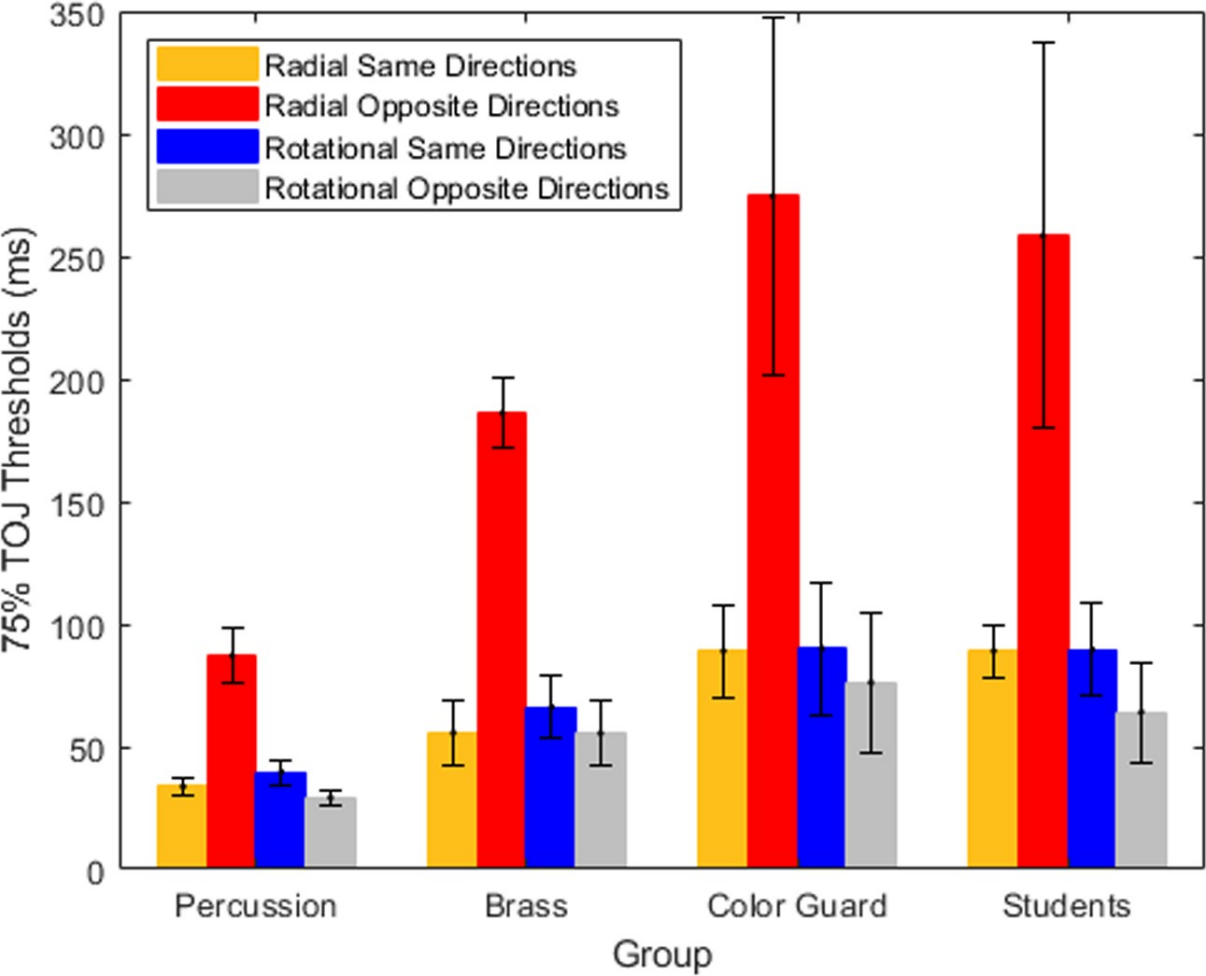
The 75% TOJ thresholds. The first three data clusters describe the performance of percussion, brass, and color guard experts in the prior study [7]. The right-most cluster describes the performance of the present study’s college students. Lower thresholds indicate better performance. Error bars reflect 1 SE from the threshold estimate of the psychometric function. For each group, changing the initial radial directions from same (gold bars) to opposite (red bars) doubled or tripled radial TOJ thresholds. By contrast, changing the initial rotational directions from same (blue bars) to opposite (gray bars) modestly reduced rotational TOJ thresholds. The present thresholds directly replicate the prior threshold-patterns [7], and most closely match the thresholds previously obtained from the color guard.

To evaluate the quantitative similarity between the present and the prior findings [7] we used Fig 4‘s psychometric functions to estimate TOJ thresholds. The estimated TOJ thresholds for Experiment 1’s participants (students) appear in Fig 5‘s right cluster. For comparison, Fig 5‘s other clusters show TOJ thresholds from the prior study’s [7] age-matched Drum Corps percussionists, brass players, and color guard. (Color guard members spin and toss flags, rifles, sabers, and batons in ways that visually interact with the musical performance.) Across stimulus conditions, all four groups show the same threshold patterns. Specifically, changing the initial radial directions from same (gold bars) to opposite (red bars) doubled or tripled radial TOJ thresholds. By contrast, changing the rotational directions from same (blue bars) to opposite (gray bars) *decreased* rotational TOJ thresholds by ~12% to ~28%. Thresholds from the present Experiment 1 (students in Fig 5‘s right cluster) best match those of the color guard from the prior study [7].

## Experiment 1: Discussion

Among college students without drum corps affiliations, Experiment 1 replicated the significant interaction effect previously observed in the TOJs of world class drum corps experts [7]. Specifically, changing the initial directions from same to opposite markedly impaired radial TOJs and modestly improved rotational TOJs. Notably, opposite initial-directions impaired radial TOJs at *each* asynchrony (±67, ±133, and ±200). This parallels the prior study, which similarly reported opposite initial-directions impairing radial TOJs at each asynchrony and for *each group*; percussion, brass, and color guard [7]. Taken together, the Motion-Type by Initial-Directions interaction has replicated across three asynchronies and four distinct populations. The effect appears reliable.

The effect also appears large. In Experiment 1, changing the initial directions from same to opposite increased radial TOJ thresholds from 89 to 258 ms. This 2.89-fold threshold elevation parallels the effect size (~2.5-fold to ~3.0-fold threshold elevation) previously observed among world class drum corps experts [7]. Insight about why TOJ thresholds doubled or tripled in the Radial-Opposite condition comes from a subtlety in Experiment 1’s results, which we consider next.

**Fig 6:**
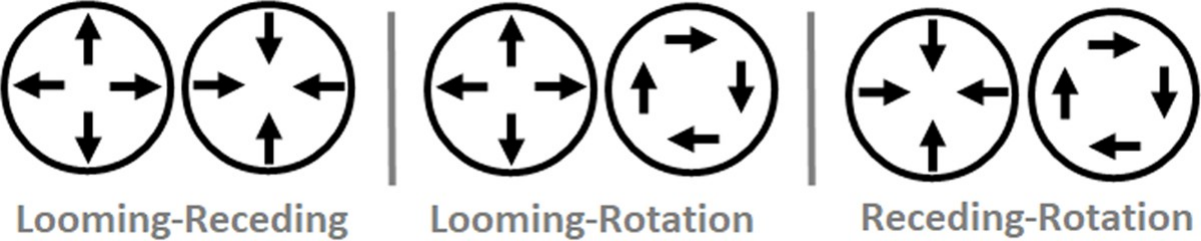
Schematic of Experiment 2 stimuli. In Experiment 2, bilaterally presented plaid pairs either loomed and receded (left), loomed and rotated (center), or receded and rotated (right) before reversing directions. Participants judged whether the left or right plaid changed direction first–a temporal order judgment (TOJ).

As noted above, in Experiment 1 Radial-Opposite TOJs often failed even at the most extreme asynchronies. To appreciate this point, consider Fig 4‘s Radial-Opposite psychometric function. The left-first responses failed to approach the “floor” and “ceiling” at the negative and positive 200 ms asynchronies, respectively. At those asynchronies, TOJ failures similarly occurred in the Radial-Opposite condition of the prior study (see Fig 6 in Matthews, Welch & Festa, 2018). Failures at the extremes of a psychometric function’s abscissa often reflect so-called “lapses”. Lapses represent performance failures attributable to stimulus-independent factors such as inattention, poor motivation, and/or motor errors. However, stimulus-independent lapses seem implausible here because we randomly interleaved Radial-Opposite trials with trials from other stimulus conditions that did not generate comparable performance failures.

An alternative explanation for the Radial-Opposite TOJ failures at the most extreme asynchronies arises from participants’ spontaneous reports about global (cross-hemifield) counter-phase depth perception. (See Introduction.) Accordingly, in Experiment 2 we tested three hypotheses about depth-related mechanisms that could have generated the observed Radial-Opposite TOJ impairments.

## Experiment 2 Mechanism identification

Table 1 summarizes the elevation in opposite-motion radial TOJ thresholds for the four groups represented in Fig 5. The threshold elevation could reflect at least three mechanisms. Here we outline these three mechanisms and their diverging predictions, which we tested in Experiment 2.

**Table 1:** Threshold elevation for opposite direction radial TOJs.

Group	Same-Direction Radial TOJ Threshold (ms)	Opposite-Direction Radial TOJ Threshold (ms)	Threshold Elevation %	Threshold Elevation Scaling Factor
Percussion Experts	34	87	158%	2.58-fold
Brass Experts	55	186	234%	3.34-fold
Color Guard Experts	89	274	208%	3.08-fold
College Students	89	258	189%	2.89-fold

Changing the plaids’ initial radial motion directions from same to opposite doubled or tripled TOJ thresholds in samples taken from four distinct populations.

### 1. Depth uncertainty (DU)

One explanation for the observed Radial-Opposite threshold elevation (Table 1) pertains to depth uncertainty. As noted above, participants reported perceiving counter-phased depth in Experiment 1’s Radial-Opposite condition. Consequently, participants in our Radial-Opposite condition had to track multiple depth planes simultaneously—a requirement unique to our Radial-Opposite condition. The other three conditions required participants to track only a single depth plane at any instant. Stated differently, trials that required instantaneously tracking a single depth plane occurred with 0.75 probability. By contrast, trials that required instantaneously tracking multiple depth planes occurred with only 0.25 probability. Because trial blocks interleaved these two trial types, participants may have adopted a perceptual set that favored the more probable single-depth-plane trials. The depth uncertainty hypothesis predicts Radial-Opposite TOJ thresholds significantly lower than in Table 1 if trials containing multiple simultaneous depth planes occur with 100% certainty.

### 2. Common fate (CF)

An alternative explanation for the observed Radial-Opposite threshold elevation (Table 1) originates from the Gestalt perceptual grouping principle of common fate. The common fate principle posits that spatially separated stimuli sharing a direction of motion become organized into a coherent perceptual unit. The plaids in our Radial-Opposite condition had no common fate cues (no shared directions). Dissimilarly, salient common fate cues occurred in each of our other three motion conditions. For example, on Radial-Same trials the two plaids initially loomed together or initially receded together. On Rotational-Same trials the two plaids initially rotated in unison either clockwise or anti-clockwise, like bicycle wheels. Less intuitively, on Rotational-Opposite trials the two plaids contained shared downward or shared upward *local* motion directions, like the shared *local* directions of interlocking gears. In short, the Radial-Opposite condition uniquely lacked the salient common-fate cues that may have otherwise facilitated perceptual organization, and TOJs. The common fate hypothesis predicts significantly elevated TOJ thresholds for any motion conditions lacking common fate cues.

### 3. Attentional prior entry (APE)

A third explanation for the observed Radial-Opposite threshold elevation (Table 1) originates from physiological and behavioral investigations of attention. Single-cell and local field potentials recorded from the primate visual cortex indicate that attention reduces neural response latencies [14, 15]. This physiological effect could generate Attentional Prior Entry (APE), the phenomenon in which observers perceive attended stimuli sooner than unattended stimuli. Several prior studies have documented these attentional effects in TOJs [16–20]. The connection between attention and TOJs seems relevant given evidence that looming (radially expanding) stimuli capture attention while receding (radially contracting) stimuli do not [21–27]. This raises the possibility that, through APE, looming stimuli become perceptually available significantly sooner than do receding stimuli. Accordingly, the APE hypothesis predicts TOJ psychometric function biases (PSE shifts) that favor initially looming stimuli. In the limit, strong APE for looming stimuli would result in erroneously classifying *all* our initially looming stimuli as having changed direction first.

## Materials and methods

### Participants

107 college-aged undergraduates who did not participate in Experiment 1 participated in Experiment 2. All had normal or corrected vision.

### Materials and apparatus

The materials and apparatus matched those of Experiment 1 except in two ways. First, because Radial-Opposite TOJ thresholds in Experiment 1 exceeded the largest tested asynchrony, we increased Experiment 2’s asynchronies to ±100, ±200, and ±300 ms. Second, we changed several of the plaid stimulus pairings (see Fig 6). Experiment 2’s Looming-Receding condition paired an initially expanding (looming) plaid and an initially contracting (receding) plaid, thereby matching Experiment 1’s Radial-Opposite trials. Experiment 2’s Looming-Rotation condition paired an initially expanding (looming) plaid with a rotating plaid. Experiment 2’s Receding-Rotation condition paired an initially contracting (receding) plaid with a rotating plaid. The Supporting Information contains sample movies (S9–S14 Movie) demonstrating these plaid pairings, with the direction changes asynchronized by 300 ms for illustration. Critically, the plaid stimulus pairs in Experiment 2 always presented multiple depth planes simultaneously, never contained common-fate motion, and always contained radial motion. Those three factors respectively permitted tests of the three above-described hypotheses; depth uncertainty, common fate, and attentional prior entry.

### Task

As in Experiment 1, participants pressed the left or right arrow key to signal whether the left or right plaid changed direction first–a TOJ. The LCD monitor visually displayed immediate accuracy feedback after each response.

### Procedure

All participants began the experiment passively viewing sample movies. Each participant then completed a 70-trial practice block that randomly interleaved asynchronies within one of Experiment 2’s stimulus conditions. After the practice block participants completed 280 analysis trials. These comprised 20 trials crossed with seven asynchronies (0, ±100, ±200, ±300 ms) and two initially looming (or receding) sides (left, right). All other aspects of the procedure matched those of Experiment 1.

### Research design & statistical analyses

Distinct participant groups completed the Looming-Receding, Looming-Rotation, and Receding-Rotation conditions. For each group, we administered the initial-looming (or receding) side variable (left, right) within participants. The dependent variables included TOJ precision and accuracy. We estimated TOJ precision (threshold) and accuracy (PSE) using Experiment 1’s psychometric function procedures and -300, -200, -100, 0, 100, 200, 300 ms asynchronies.

In addition to these group-level descriptive statistics, we also computed inferential statistics. This began by using the above-described psychometric function procedure to determine the TOJ threshold and PSE for each participant in Experiments 1 and 2. Shapiro-Wilk tests indicated that the resulting TOJ threshold and PSE distributions violated the normality assumption that underlies parametric tests. Consequently, non-parametric Mann-Whitney tests evaluated TOJ-threshold differences across groups from Experiments 1 and 2. Similarly, non-parametric Wilcoxen tests evaluated within-participant PSE-shifts arising from Experiment 2’s two initially looming (or receding) sides (left, right). For each Mann-Whitney and Wilcoxen non-parametric inferential statistic we determined a non-parametric rank sum Z-score as a dimensionless indicator of effect size [28]. For completeness, we additionally quantified effect size in milliseconds for TOJ thresholds and PSEs.

### Inclusion / exclusion criteria

The statistical analyses included data from each participant whose TOJ performance statistically exceeded chance (binomial test p<0.001). This inclusion criterion minimally required 60% correct TOJ performance across the 240 non-zero asynchrony trials for analysis. (The remaining 40 zero-asynchrony trials for analysis provided no basis for objectively determining a correct TOJ.) This criterion resulted in excluding data from 31 participants. Data from the remaining 76 (71% of 107) participants appear in the Results. As noted above, the Open Science Framework [https://osf.io/d72bv/] contains the raw data from *all* participants.

## Experiment 2: Results

### Depth uncertainty (DU) hypothesis

**Fig 7:**
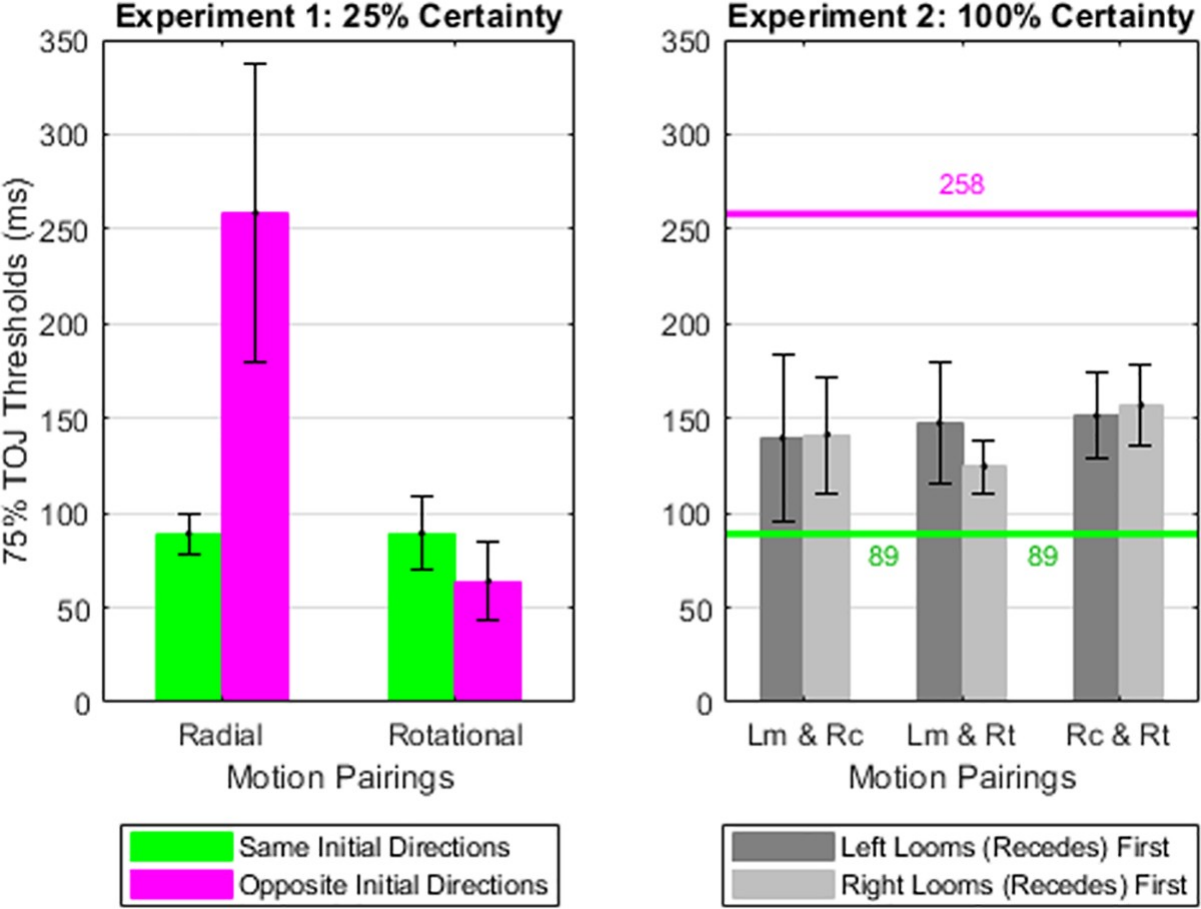
TOJ thresholds from Experiments 1 and 2. Both the Directional Uncertainty (DU) and the Common Fate (CF) hypotheses predict the TOJ threshold pattern in Experiment 1 (left panel). The DU and CF hypotheses make diverging falsifiable predictions for Experiment 2 (right panel). TOJ thresholds at or above the magenta horizontal line (258 ms) would disconfirm DU predictions because multiple simultaneous depths occurred on all Experiment 2 trials. Experiment 2’s TOJs (right panel, gray bars) fell significantly (100 ms or more) below the DU disconfirmation line. The significant (100+ ms) threshold reduction between Experiment 1’s Radial-Opposite condition and Experiment 2 demonstrates that depth certainty can improve motion TOJ thresholds. TOJ thresholds at or below the green horizontal line (89 ms) would disconfirm CF predictions because Experiment 2’s stimuli contained no common fate. Experiment 2’s TOJs consistently exceeded the CF (89 ms) disconfirmation line, though by non-significant margins after Bonferroni corrections. The consistent (albeit non-significant) tendency toward elevated thresholds suggests that common fate motion contributed to the lower (< = 89 ms) TOJ thresholds in Experiment 1. Error bars reflect one standard error of the threshold estimate. Abbreviations: In condition Lm & Rc, one plaid initially loomed while the other initially receded. In condition Lm & Rt, one plaid initially loomed while the other initially rotated. In condition Rc & Rt one plaid initially receded while the other initially rotated.

The Depth Uncertainty (DU) hypothesis predicts large TOJ threshold elevations when cues to multiple simultaneous depth planes occur infrequently. This would explain the threshold spike in Experiment 1’s Radial-Opposite condition (Fig 7, left panel), where multiple simultaneous depth planes occurred with just 25% probability. The DU hypothesis also predicts TOJ thresholds significantly lower than those in Experiment 1 if stimuli presenting multiple simultaneous depth planes occur with certainty. Accordingly, Fig 7 contains a DU “disconfirmation line” (magenta horizontal line at 258 ms, Fig 7‘s right panel). TOJ thresholds at or above that line in Experiment 2 would disconfirm the DU hypothesis because *all* Experiment 2 stimuli contained multiple simultaneous depth planes.

Fig 7‘s right panel indicates that Experiment 2’s conditions uniformly generated TOJ thresholds at least 100 ms lower (better) than the DU disconfirmation line. The performance differences between Experiment 1’s Radial-Opposite condition and Experiment 2 suggest that directional certainty conferred a threshold advantage between 101 and 133 ms. Critically, Experiment 2’s Looming-Receding condition produced thresholds 117 ms (45%) lower than Experiment 1’s Radial-Opposite condition, despite depth cues remaining *identical* across those two conditions. Bonferroni-corrected Mann-Whitney tests confirmed that Experiment 2’s six conditions each produced significantly (p<0.05) lower threshold-ranks than did Experiment 1’s Radial-Opposite condition. Table 2 (top) summarizes those statistical comparisons. The effect sizes in Table 2 reveal that Experiment 2’s directional certainty reduced mean threshold ranks by between 2.83 and 3.54 rank sum Z-scores. Overall, the data demonstrate that depth certainty reliably reduced motion-defined TOJs.

**Table 2:** Threshold-Ranks from Experiments 1 and 2 –Inferential Statistics.

Experiments	Condition	N Exp1 / Exp 2	Mean Threshold Ranks Exp 1 / Exp 2	Mann-Whitney P-Value	Effect Size Rank Sum Z-Score
Exp 1 Radial-Opposite vs Exp 2 Looming-Receding	Left 1st	31 / 26	35.32 / 21.46	0.002*	-3.14
	Right 1st		35.65 / 21.08	0.001*	-3.30
Exp 1 Radial-Opposite vs Exp 2 Looming-Rotation	Left 1st	31/ 22	32.06 / 19.86	0.005*	-2.83
	Right 1st		33.32 / 18.09	<0.001*	-3.54
Exp 1 Radial-Opposite vs Exp 2 Receding-Rotation	Left 1st	31 /28	36.65 / 22.64	0.002*	-3.13
	Right 1st		37.03 / 22.21	0.001*	-3.31
Exp 1 Radial-Same vs Exp 2 Looming-Receding	Left 1st	31 / 26	25.45 / 33.23	0.078	-1.76
	Right 1st		25.39 / 33.31	0.073	-1.79
Exp 1 Radial-Same vs Exp 2 Looming-Rotation	Left 1st	31/ 22	22.77 / 32.95	0.018	-2.37
	Right 1st		23.58 / 31.82	0.056	-1.91
Exp 1 Radial-Same vs Exp 2 Receding-Rotation	Left 1st	31 / 28	24.71 / 35.86	0.013	-2.49
	Right 1st		24.97 / 35.57	0.018	-2.37

(Top three rows) Each of Experiment 2’s six conditions generated significantly lower (finer) threshold-ranks than those from Experiment 1’s Radial-Opposite condition. Asterisks indicate that these statistical comparisons remained significant after Bonferroni correction. The rank-sum Z-scores ranged between -2.83 and -3.54, relatively large effect sizes that support the Directional Uncertainty (DU) hypothesis. (Bottom three rows) Each of Experiment 2’s six conditions generated higher (worse) threshold-ranks than those from Experiment 1’s Radial-Same condition. None of these statistical comparisons remained significant after Bonferroni correction. The rank-sum Z-scores ranged between -1.76 and -2.49, relatively smaller effect sizes that collectively indicate modest support for the Common Fate (CF) hypothesis.

### Common fate (CF) hypothesis

The Common Fate (CF) hypothesis posits that spatially separated stimuli sharing a direction of motion become organized into a coherent perceptual unit. This Gestalt law of perceptual organization predicts high or low TOJ thresholds for stimulus pairs that respectively lack or share common fate motion. This correctly predicts the TOJ threshold pattern observed in Experiment 1 –displayed for comparison in Fig 7‘s left panel. The CF hypothesis also posits that participants in Experiment 1 achieved TOJ thresholds at or below 89 ms by exploiting common fate motion. If so, one would expect thresholds consistently greater than 89 ms in Experiment 2, which contained no common fate motion. Accordingly, Fig 7 contains a CF “disconfirmation line” (green horizontal line at 89 ms, Fig 7‘s right panel). TOJ thresholds observed at or below that line in Experiment 2 would demonstrate the non-necessity of common fate cues for reaching Experiment 1’s performance levels.

Fig 7‘s right panel indicates the Experiment 2’s conditions uniformly generated TOJ thresholds at least 35 ms (39%) larger (worse) than the CF disconfirmation line. The performance differences between Experiment 1’s Radial-Same condition and Experiment 2 suggest common fate conferred a threshold advantage between 36 and 68 ms. Intriguingly, Experiment 1’s Radial-Same stimuli generated lower TOJ thresholds than did Experiment 2’s conditions, even though Experiment 1’s Radial-Same stimuli occurred with only 25% probability. Stated differently, common-fate motion with less than 100% probability generated better TOJ thresholds than did stimuli lacking common fate motion with 100% probability. Mann-Whitney tests revealed marginally higher thresholds in each of Experiment 2’s conditions than in Experiment 1’s Radial-Same condition; p-values initially ranged between 0.013 and 0.078. However, none of the six comparisons remained significant after Bonferroni correction (see Table 2, bottom). The effect sizes in Table 2 (bottom) indicate that Experiment 1’s common fate reduced mean threshold ranks by between 1.76 and 2.49 rank sum Z-scores. Overall, the data demonstrate a tendency for common fate to reduce motion-defined TOJs, albeit a smaller effect than that of depth certainty.

### 3. Attentional prior entry (APE) hypothesis

The Attentional Prior Entry (APE) hypothesis posits that looming stimuli become perceptually available sooner than do receding stimuli, which capture less attention [21–27]. Attentional prior entry therefore predicts PSE biases favoring initially looming stimuli in Experiment 2’s psychometric functions.

**Fig 8:**
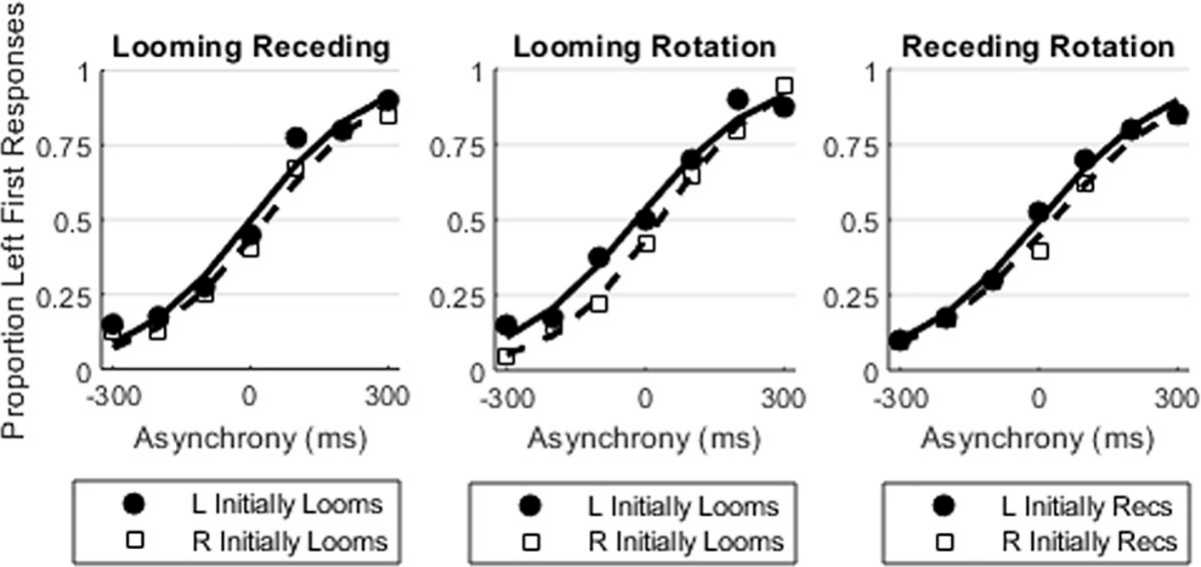
Psychometric Functions from Experiment 2. In the Looming-Receding and Looming-Rotation conditions, solid circles and solid lines correspond to initially looming stimuli on the left side. Trials with left-initially-looming stimuli shifted psychometric functions modestly leftward relative to right-initially-looming trials (open squares, dotted lines). Likewise, in the Receding-Rotation condition, trials with left-initially-receding stimuli shifted psychometric functions modestly leftward relative to right-initially-receding trials.

Experiment 2’s psychometric functions appear in Fig 8. For the Looming-Receding and Looming-Rotation conditions, solid circles and solid lines reflect trials on which the left plaid initially loomed. Open squares and dotted lines reflect trials with an initially looming right plaid. Changing the initially looming side from left to right shifted the psychometric function modestly rightward in the Looming-Receding and Looming-Rotation conditions. These rightward psychometric-function shifts reached statistical significance (Wilcoxen Tests, p<0.05), and corresponded to modestly larger right than left PSE values (see Table 3).

**Table 3:** Experiment 2’s Inferential Statistics and PSE values.

Experimental Condition	Right 1^st^ PSE Minus Left 1^st^ PSE Negative / Positive	Wilcoxen Signed Ranks P-Value	Effect Size Rank Sum Z-Score	Left 1^st^ PSE (ms)	Right 1^st^ PSE (ms)
Looming Receding (n = 26)	8 / 18	0.019*	-2.35	+0.82	+32.89
Looming Rotation (n = 22)	7 / 15	0.046*	-2.00	-18.30	+31.01
Receding Rotation (n = 28)	9 / 19	0.084	-1.73	+1.33	+31.37

In Experiment 2’s Looming-Receding and Looming-Rotation conditions, the Point of Subjective Equality (PSE) increased modestly after changing the initially looming side from left to right. These PSE shifts reflect a small fraction of the values that Attentional Prior Entry (APE) would need to significantly elevate TOJ threshold estimates. Similarly modest PSE increases also occurred in the Receding-Rotation condition when the initially *receding* side switched from left to right. Asterisks indicate significant (p<0.05) PSE shifts according to non-parametric Wilcoxen signed-ranks tests.

While the direction of these shifts matches the APE hypothesis’ predictions, the magnitude of the shifts does not. Specifically, the PSEs shifted by amounts too small to generate systematic TOJ errors at even our briefest (±100 ms) asynchronies. Moreover, a comparably modest rightward PSE shift also occurred in the Receding-Rotation condition after changing the initially *receding* side from left to right. (See Fig 8‘s right panel, and Table 3‘s right columns.) This differs from the prior finding that looming (radially expanding) stimuli capture attention while receding (radially contracting) stimuli do not [21–27]. Overall, Experiment 2’s small and similar PSE shifts for looming and receding stimuli indicate that APE did not cause Experiment 1’s radial-opposite TOJ threshold elevation.

## Experiment 2: Discussion

In Experiment 2 we tested three hypotheses for the reliable and large threshold elevation associated with Radial-Opposite TOJs. Experiment 2 provided support for the Depth Uncertainty hypothesis. When the probability of trials containing multiple simultaneous depth planes changed from 25% to 100%, TOJ thresholds improved (decreased) by more than 100 ms. Experiment 2 also provided support for the Common Fate hypothesis, albeit a smaller effect size than for depth uncertainty. Specifically, differencing the results from Experiments 1 and 2 revealed that common fate motion cues conferred a threshold advantage between 36 and 68 ms. By contrast, Experiment 2’s data disconfirmed the PSE shifts predicted by the Attentional Prior Entry hypothesis. Although important in other contexts [21–27], attentional prior entry does not explain Experiment 1’s Radial-Opposite TOJ threshold elevation.

The finding that radial-opposite TOJ thresholds improved with increasing depth-certainty relied on increasing *motion* certainty. Specifically, we controlled depth-certainty by experimentally controlling the certainty of radial expansion, radial contraction, and rotational motion, i.e., two-dimensional motion. Our findings therefore extend those of earlier studies that similarly generated performance improvements by increasing the certainty of one-dimensional (linear) motion [29–31].

Experiment 2’s 100% certainty about motion and depth still failed to improve performance to the level attained with Experiment 1’s less probable common fate motion. This performance gap points to the distinct depth requirements in the two experiments. Experiment 1’s common-fate-motion conditions (Radial-Same, Rotational-Same, Rotational-Opposite) never required tracking multiple depth planes simultaneously, whereas all of Experiment 2’s conditions did. Depth-plane *multiplicity* differs from depth-plane *certainty*.

Taken together, Experiments 1 and 2 demonstrate that depth-plane multiplicity impaired TOJs even when depth-plane certainty improved TOJs. Previous visual search studies that manipulated binocular rather than monocular depth cues similarly reported a cost for tracking multiple depth planes simultaneously. Specifically, those studies found slowed reaction times for detecting targets among distractors at distinct stereoscopically defined depths [32, 33].

Experiment 2 also revealed a global-to-local (coarse-to-fine) *spatial* sequence in visual *timing* sensitivity. This follows because manipulating global (cross-hemifield) depth information generated large TOJ threshold variations while local (within hemifield) linear motion vectors remained constant.

## General discussion

Competing theories of perceptual learning in drum corps experts motivated a previous study on visual temporal order judgments (TOJs) [7]. That theoretically-driven study generated a surprising observation. Relative to TOJ thresholds from other radial and rotational motion combinations, Radial-Opposite TOJ thresholds doubled or tripled. This empirical observation drove the present study. Specifically, we investigated the ironic possibility of global-direction reversals impairing sensitivity to the timing of their constituent local-direction reversals.

The present Experiment 1 directly replicated the previous finding [7] that global-direction reversals doubled or tripled radial TOJ thresholds without elevating rotational TOJ thresholds. This direct replication demonstrates the effect’s reliability, now shown at various temporal asynchronies and in four distinct populations. These include percussion experts, brass experts, color guard experts, and college students, with each group’s Radial-Opposite TOJ thresholds doubling or tripling–a large effect size. Critically, the effect appears to depend on *perceived* depth. Insight about the role of perceived depth came from participants’ spontaneous, qualitative verbal reports and hand gestures. These described the Radial-Opposite stimuli as “…moving in and out”, counter-phased in depth across hemifields.

Building on the participants’ qualitative insights, we conducted Experiment 2 to quantitatively evaluate how the Radial-Opposite threshold elevation depends on perceived depth. We found little evidence that the effect reflects attentional prior entry for stimuli looming in depth. Dissimilarly, depth certainty and common-fate-depth-motion each altered TOJs. Depth certainty improved (reduced) Radial-Opposite TOJ thresholds by 100+ ms compared to a condition having less depth certainty but otherwise identical Radial-Opposite directions. Evidence for a more modest influence of common-fate-depth-motion came from contrasting Experiment 1’s Radial-Same and Experiment 2’s Looming-Receding conditions. Both conditions required tracking multiple depth planes. However, Experiment 1’s Radial-Same condition occurred with 25% probability and required tracking stimuli that loomed synchronously or receded synchronously across hemifields. By contrast, Experiment 2’s Looming-Receding condition required tracking stimuli that loomed in one hemifield and receded in the other–with 100% certainty. Despite this 100% depth certainty, Experiment 2’s Looming-Receding condition generated TOJ thresholds 35+ ms higher (worse) than those of Experiment 1’s less probable Radial-Same condition. Likewise, Experiment 2’s Looming-Rotation and Receding-Rotation conditions each generated TOJ thresholds 35+ ms higher (worse) than those of Experiment 1’s less probable Radial-Same condition. Taken together, Experiment 2 demonstrated that participants struggled to simultaneously track distinct left- and right-hemifield depth planes.

**Fig 9:**
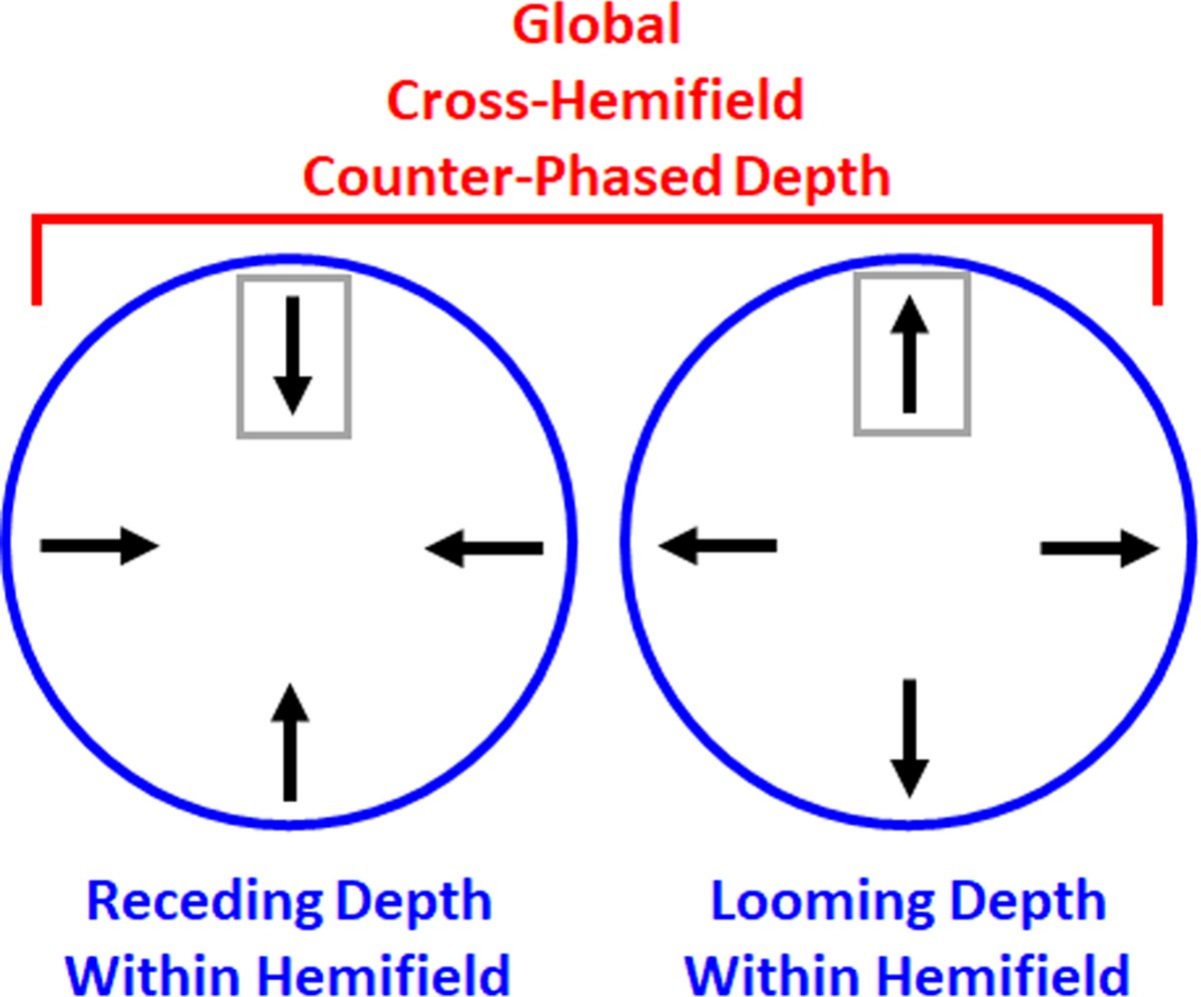
Emergent motion and the relational nature of TOJs. Counter-phased depth cues in the present study emerged bilaterally from the radial motion differences between the hemifields (circles). The distinct radial motion in each hemifield emerged from appropriately arranged local linear motion components (arrows). Differencing direction-reversal-times from two local linear motion components, one sampled from each hemifield (gray rectangles), would suffice for the present TOJs. Altering the *spatial* (depth) phase relationships between the left and right hemifields altered *temporal* order judgments (TOJs).

Fig 9 illustrates one example of the stimuli that required tracking distinct left- and right-hemifield depth planes for the present TOJs. In principle, the present TOJs merely required differencing direction-reversal-times of two local linear motion signals; one sampled from each hemifield’s plaid (gray rectangles). The local linear motion signals (arrows) generated a two-dimensional radial motion pattern within each hemifield. Spontaneous verbal reports and hand gestures indicated that participants perceived these bilateral radial motion patterns as looming in one hemifield and receding in the other. The perception of bilaterally distinct depth motion masked local direction-reversal-times and degraded participants’ TOJ performance. These results establish that local motion *time* sampling can depend on *spatially* global relationships. This follows because TOJs varied markedly across conditions comprising the same local motion components, spatially rearranged to generate distinct depth relationships across hemifields.

The finding that distinct depth planes across hemifields doubled or tripled TOJ thresholds may seem surprising given earlier reports about bilateral task performance. Specifically, several studies have revealed independent left and right hemifield resources for attentionally tracking objects [34] and elementary visual features [35, 36]. Independent bilateral resources would lead one to predict, incorrectly, that our bilaterally distinct depth planes would not elevate TOJ thresholds. By contrast, the observed TOJ-threshold elevation matches predictions that follow from perceptual grouping experiments. These studies demonstrate that perceptual grouping occurs *less* efficiently across hemifields (bilaterally) than within hemifields (unilaterally) [37, 38]. Grouping likely influenced the present TOJ thresholds, which increased and decreased respectively in the absence and presence of common fate motion–a powerful grouping cue.

Removing common fate grouping cues from Experiment 2’s stimuli increased TOJ thresholds to 125 ms or more. This comparatively sluggish temporal precision matches what one might expect from so-called “attention-based” motion perception [39, 40]. Attention-based motion perception can support accurate speed or direction judgments for features that do not stimulate motion-detecting neurons in the early visual pathway. In principle, the direction reversals in our bilaterally presented plaid stimuli constitute features that could generate leftward or rightward attention-based motion cues for TOJs. To the extent that stimuli lacking common-fate grouping cues require more attentional resources, one would expect higher TOJ thresholds -as observed here. These higher thresholds could reflect an increase in attentional lapses, visual attention’s comparatively poor temporal resolution, or both. Previous estimates of attention’s temporal resolution range between 4 and 10 Hz [41–45], i.e., 100–250 ms temporal periods. Those temporal periods better match TOJ thresholds obtained here in the absence (125–258 ms) than presence (64–89 ms) of common fate grouping cues.

## Conclusions

**Fig 10:**
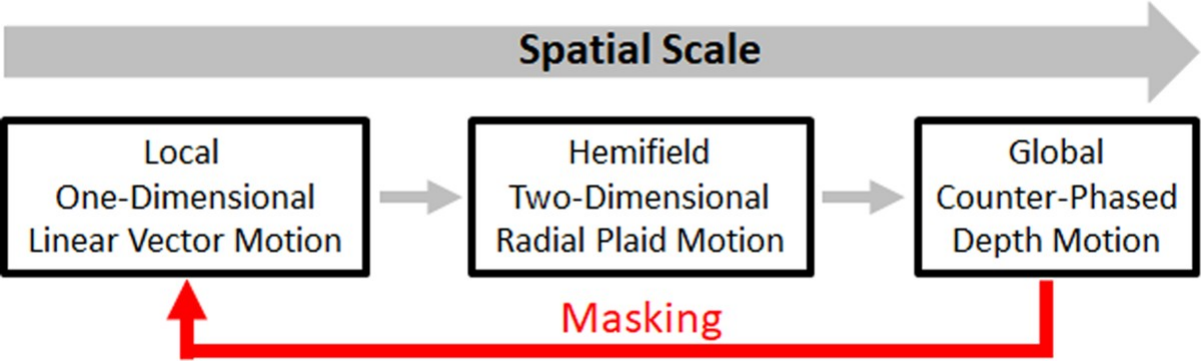
Coarse-to-fine spatiotemporal masking model. Global, counter-phased depth perception ironically impairs sensitivity to direction-reversal-times among its constituent local linear motion signals (red arrow).

Overall, our experiments demonstrate that global depth perception alters local timing sensitivity. The doubling or tripling of TOJ thresholds for our bilaterally presented stimuli reveal coarse-to-fine influences across spatial scales. Fig 10 schematizes this coarse-to-fine sequence. One-dimensional linear vectors at the smallest spatial scale (left) generated two-dimensional radial motion at an intermediate spatial scale; the plaid within each hemifield (center). In turn, opposite radial plaid directions in the left and right hemifields generated counter-phased depth motion at the most global (cross-hemifield) spatial scale. The global (cross-hemifield) counter-phased depth motion subsequently masked information about the *timing* of local direction reversals (red arrow).

More broadly, the coarse-to-fine spatial influences on the present TOJ task align with the coarse-to-fine spatial influences demonstrated for diverse visual phenomena. These phenomena include stereoscopic depth perception [46–49], hysteresis in motion perception [50], brightness illusions [51, 52], and "the dress" color illusion [53]. Moreover, our results extend the recent coarse-to-fine motion demonstrations in which global direction reversals masked local color and orientation changes [5, 6]. We find that global direction reversals can ironically mask the temporal properties of their own, local direction components.

## Supporting information

S1 Movie Experiment 1’s Radial-Same stimuli, with left-first direction change. (MP4)

S2 Movie Experiment 1’s Radial-Same stimuli, with right-first direction change. (MP4)

S3 Movie Experiment 1’s Radial-Opposite stimuli, with left-first direction change. (MP4)

S4 Movie Experiment 1’s Radial- Opposite stimuli, with right-first direction change. (MP4)

S5 Movie Experiment 1’s Rotational-Same stimuli, with left-first direction change. (MP4)

S6 Movie Experiment 1’s Rotational-Same stimuli, with right-first direction change. (MP4)

S7 Movie Experiment 1’s Rotational-Opposite stimuli, with left-first direction change. (MP4)

S8 Movie Experiment 1’s Rotational-Opposite stimuli, with right-first direction change. (MP4)

S9 Movie Experiment 2’s Looming-Receding stimuli, with left-first direction change. (MP4)

S10 Movie Experiment 2’s Looming-Receding stimuli, with right-first direction change. (MP4)

S11 Movie Experiment 2’s Looming-Rotation stimuli, with left-first direction change. (MP4)

S12 Movie Experiment 2’s Looming-Rotation stimuli, with right-first direction change. (MP4)

S13 Movie Experiment 2’s Receding-Rotation stimuli, with left-first direction change. (MP4)

S14 Movie Experiment 2’s Receding-Rotation stimuli, with right-first direction change. (MP4)
